# Revisiting aortic valve prosthesis choice in patients younger than 50 years: 10 years results of the AUTHEARTVISIT study

**DOI:** 10.1093/ejcts/ezad308

**Published:** 2023-09-26

**Authors:** Denise Traxler, Pavla Krotka, Berthold Reichardt, Dragan Copic, Cecilia Veraar, Michael Mildner, Ralph Wendt, Johann Auer, Julia Mascherbauer, Hendrik Jan Ankersmit, Alexandra Graf

**Affiliations:** Clinic of Thoracic Surgery, Medical University of Vienna, Austria; Laboratory for Cardiac and Thoracic Diagnosis, Regeneration and Applied Immunology, Austria; Department of Oral and Maxillofacial Surgery, Medical University of Vienna, Austria; Center for Medical Data Science, Medical University of Vienna, Austria; Austrian Social Health Insurance Fund, Eisenstadt, Austria; Clinic of Thoracic Surgery, Medical University of Vienna, Austria; Laboratory for Cardiac and Thoracic Diagnosis, Regeneration and Applied Immunology, Austria; Division of Nephrology and Dialysis, Medical University of Vienna, Austria; Laboratory for Cardiac and Thoracic Diagnosis, Regeneration and Applied Immunology, Austria; Division of Cardiothoracic and Vascular Anesthesia and Intensive Care Medicine, Medical University of Vienna, Austria; Department of Dermatology, Medical University of Vienna, Austria; Department of Nephrology, St. Georg Hospital, Leipzig, Germany; Department of Internal Medicine I with Cardiology and Intensive Care, St. Josef Hospital Braunau, Braunau am Inn, Austria; Department of Internal Medicine 3, University Hospital St. Poelten, Austria; Clinic of Thoracic Surgery, Medical University of Vienna, Austria; Laboratory for Cardiac and Thoracic Diagnosis, Regeneration and Applied Immunology, Austria; Center for Medical Data Science, Medical University of Vienna, Austria

**Keywords:** Aortic valve replacement, Mechanical aortic valve prosthesis, Bioprosthesis

## Abstract

**OBJECTIVES:**

This population-based cohort study investigated mid-term outcome after surgical aortic valve replacement with a bioprosthetic or mechanical valve prosthesis in patients aged <50 years in a European social welfare state.

**METHODS:**

We analysed patient data from the main social insurance carriers in Austria (2010–2020). Subsequent patient-level record linkage with national health data provided patient characteristics and clinical outcome. Survival, reoperation, myocardial infarction, heart failure, embolic stroke or intracerebral haemorrhage, bleeding other than intracerebral haemorrhage and major adverse cardiac events were evaluated as outcomes.

**RESULTS:**

A total of 991 patients were analysed. Regarding demographics, no major differences between groups were observed. Multivariable Cox regression revealed no significant difference in overall survival (*P* = 0.352) with a median follow-up time of 6.2 years. Reoperation-free survival was decreased (hazard ratio = 1.560 [95% CI: 1.076–2.262], *P* = 0.019) and the risk for reoperation was increased (hazard ratio = 2.770 [95% CI: 1.402–5.472], *P* = 0.003) in patients who received bioprostheses. Estimated probability of death after reoperation was 0.23 (CL: 0.08–0.35) after 2 years and 0.34 (CL: 0.06–0.53) after 10 years over both groups. Regarding further outcomes, no significant differences between the two groups were observed.

**CONCLUSIONS:**

In patients below 50 years of age receiving aortic valve replacement, implantation of bioprostheses when compared to mechanical heart valve prostheses was associated with a significantly higher rate of reoperations and reduced reoperation-free survival. Nevertheless, we could not observe a difference in overall survival. However, long-term follow-up has to evaluate that a significantly lower rate of reoperations may translate in consistently improved long-term survival.

**Figure:**
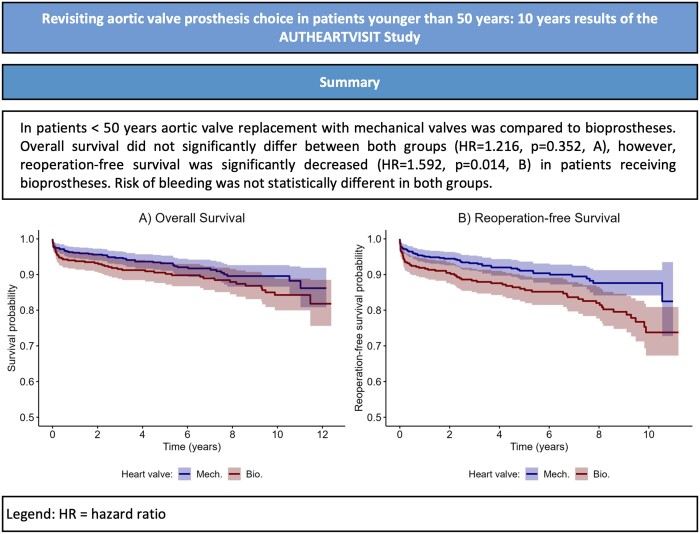

## INTRODUCTION

Surgical aortic valve replacement (sAVR) represents the standard treatment option for severe aortic valve disease and is performed in ∼280 000 patients annually, with this number projected to rise. [1] Current guidelines of the European Society of Cardiology (ESC) advise a preference for mechanical aortic valve prosthesis (sM-AVR) in patients <65 years [2]. In contrast, the American Heart Association/American College of Cardiology (AHA/ACC) guideline states that it is reasonable to implant a bioprosthesis (sB-AVR) in patients aged >50 years [3]. These AHA/ACC guidelines formulate their advice by utilizing multiple propensity score analyses that imply that age and choice of prosthesis have no effect on morbidity and survival [4].

These conclusions do not correspond with reports published in the last decade. Various studies [5–7] have supported the notion that the implantation of sB-AVR versus sM-AVR may have dire consequences in relation to mortality and morbidity in patients below 65 years. An independent hazard ratio (HR) meta-analysis publication has confirmed this. This study analysed randomized controlled trials and observational studies, using propensity score matching and inverse probability weighting to examine the clinical outcomes of patients receiving sB-AVR or sM-AVR <70 years of age. They conclude that sM-AVR has a significant survival benefit as compared to sB-AVR. Of interest was the finding that showed that survival rates were not influenced by the type of AV prosthesis in patients younger than 50 years [8].

To evaluate the effect of sM-AVR or sB-AVR on morbidity and mortality in patients below 50 years, we conducted a population-based study including about 1000 patients who underwent primary isolated sAVR in a well-organized European welfare state between 2010 and 2020. The health care in Austria is a national health care system with good access to care, few malpractice lawsuits and little tendency towards overuse of medical resources. Approximately 98% of the Austrian population is registered in the public health insurance system; however, a minority of patients paying for medical supplies using private insurance cannot be included in this AUTHEARTVISIT investigation.

The primary objective was to compare mid-term mortality between sB-AVR and sM-AVR. Further objectives were to compare reoperation risk as well as the risk of developing myocardial infarction, embolic stroke or intracerebral haemorrhage (ICH), heart failure (HF), bleeding other than ICH and combined major adverse cardiac events (MACE) after sAVR.

## MATERIALS AND METHODS

### Ethical statement

This study complies with the Declaration of Helsinki and was approved by the Ethics Committee of Lower Austria (GS1-EK-4/722-2021). Furthermore, this trial was registered with clincialtrials.gov (NCT05627973). Due to the retrospective nature of this study, written informed consent was not obtained.

### Study design

This study was a national population-based cohort study. Study data were generated retrospectively by retrieving data from main social insurance carriers in Austria. Clinical and operative data of all patients registered in the Austrian Health Care System who underwent sAVR (either using a mechanical replacement—MEL code DB082—or a bioprosthetic replacement—MEL codes DB060, DB070 and DB080) in Austria (years 2010–2020) at an age below 50 years were obtained. Patients aged <18 years, receiving transcatheter aortic valve replacement (TAVR), patients with concomitant heart surgery or receiving a coronary artery stent within 4 months prior to aortic valve replacement (AVR) were excluded from this analysis. We selected a period of 4 months previous to the surgery for exclusion of patients with percutaneous coronary intervention to guarantee selection of patients with pure AVR procedures (see Supplementary Material).

For each patient, billing information (based on MEL codes) and diagnoses [based on International Statistical Classification of Diseases and Related Health Problems in its 10th revision (ICD-10) codes] were available from 1 year before index-op up to study closure. To evaluate outcomes for each patient, data were scanned from index-op to study closure for the corresponding ICD-10 or MEL codes. Data available 1 year before index-op were scanned for each patient to evaluate possible comorbidities. Detailed descriptions of all ICD-10 and MEL codes used for the outcome and comorbidity variables are shown in Supplementary Material, Tables S1–S5. Note that the ICD-10 in the version of 2019, available on https://icd.who.int/browse10/2019/en, was used.

### Outcomes

Primary outcome was overall survival after AVR. The outcome reoperation was defined as the first reconstruction or replacement of any heart valve after the aortical valve replacement (MEL codes DB020–DB142). To evaluate reoperation-free survival, the combined end point consisting of death and reoperation was used. Further outcomes were embolic stroke or ICH (ICD-10 Codes I.61x, I.63x, I.64, G.45x), myocardial infarction (I21.x), HF (I11.0, I13.0, I13.2, I50, I50.0, I50.1, I50.9), major bleeding events other than ICH (I600–I608, I850, I982, I983, K250–K296, K922, N421, R041, R048, R049, R58, S064, T810) and MACE. MACE was defined as a combined end point consisting of death, reoperation, HF, myocardial infarction and embolic stroke or ICH (ICD-10 codes equal those as stated above for each single end point). Details of all definitions are found in the Supplementary Material.

### Statistical analysis

Continuous data are presented descriptively as median with interquartile range (IQR), while categorical data are shown as counts and percentages. Categorical variables were compared between both groups using chi-squared test.

For survival as well as reoperation-free survival and MACE, Cox regression and Kaplan–Meier curves were used to evaluate the benefit of 1 valve type (sM-AVR versus sB-AVR). For multivariable analysis, the following covariables were additionally included as possible confounders to the statistical model: age (in years), sex (female versus male), HF, myocardial infarction, embolic stroke or ICH, diabetes mellitus, adiposity, hyperlipidaemia, hyperuricaemia/gout, cardiomyopathies, valvular, hypertensive, inflammatory and rhythmogenic cardiopathies, ischaemic heart disease, atherosclerosis, pulmonary diseases and kidney diseases prior to operation. These comorbidities were defined using ICD-10 codes available 1 year before index-op (see Supplementary Material).

Proportional hazard assumption was evaluated using Schönfeld residuals, collinearity was evaluated using variance inflation factors.

The end points reoperation, HF, myocardial infarction, embolic stroke or ICH and bleeding other than ICH were evaluated by competing risk analysis using death as competing event. For all analyses, HR and survival probabilities as well as corresponding 95% confidence intervals are presented. Note that for the end points MACE and HF, patients with a diagnosed HF before the surgery were excluded to focus on newly diagnosed events after surgery. HR are presented as sB-AVR versus sM-AVR; thus, an HR of larger than 1 indicates an increased probability of the corresponding event in the bioprostheses group (and vice versa).

Statistical analyses were carried out and graphs were generated in R (version 4.1.3) using the following packages: survival (version 3.2.13), survminer (version 0.4.9) and cmprsk (2.2.11). All tests were performed at a two-sided significance level of 0.05.

## RESULTS

### Study population and patient characteristics

A total of 991 patients were included in this analysis: 592 (59.7%) patients received sM-AVR and 399 (40.3%) received sB-AVR.

The median age at index operation was 43 years (IQR: 35–46 years). The median age at mechanical valve replacement was 43 years (IQR: 36–46 years) and 42 years (IQR: 34–47 years) at implantation of bioprostheses. The sM-AVR group compiled 461 (77.9%) male and 131 (22.1%) female patients. In the sB-AVR cohort 290 (72.7%), patients were male and 109 (27.3%) were female.

**Figure 1:**
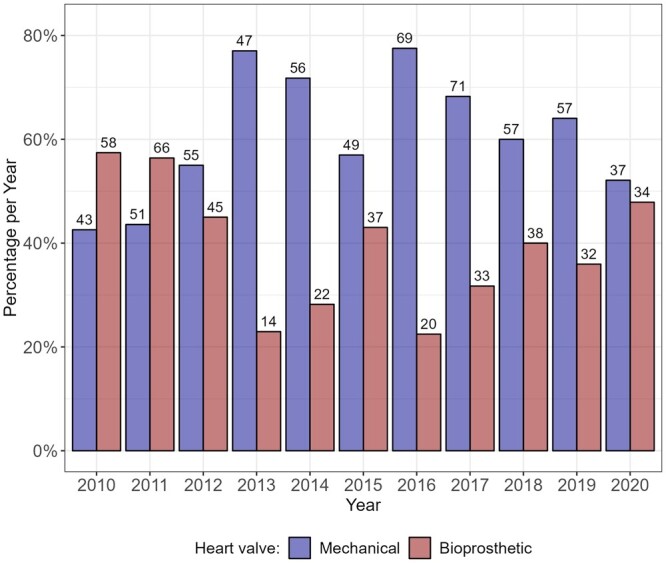
Distribution of Heart Valve Types within the study period. Percentages per year (displayed on the *y*-axis) and absolute numbers (shown above the bars) of patients receiving mechanical aortic valve or bioprosthetic valve replacement per calendar year.

Figure 1 presents the number of patients per group and calendar year of operation. Over the years, both valve types were used for patients younger than 50 years. In 2020, both valve types were used equally in our study population. For details on different valve types, see also Supplementary Material, Table S6.

Only a small number of patients had pre-existing medical diagnoses; those data are given in Supplementary Material, Table S7. The only exceptions were valvular or other cardiopathies, which were present in most of the patients. Patients receiving bioprostheses had a higher rate of liver disease (*P* = 0.03) prior to the index operation. In contrast, patients in the sM-AVR group were diagnosed more often with aortic disease (*P* = 0.03).

A similar picture was reflected by medication prior to the index operation. However, no significant between-group difference was detected (Supplementary Material, Table S8).

### Survival and further outcome parameters

Overall, 99 patients died within the follow-up period, among whom 50 patients received mechanical AVR (8.4% of all sM-AVR patients) and 49 received bioprostheses (12.3% of all sB-AVR patients). Sixty-nine patients died during a hospital stay (38 patients in the sM-AVR and 31 in the sB-AVR group). Out of these 38 patients in the sM-AVR group, 22 (57.9%) patients were reported with cardiovascular reasons as main diagnoses for the corresponding hospital stay. Out of the 31 patients in the sB-AVR group, 24 (77.4%) patients were reported with cardiovascular reasons as main diagnoses.

**Figure 2:**
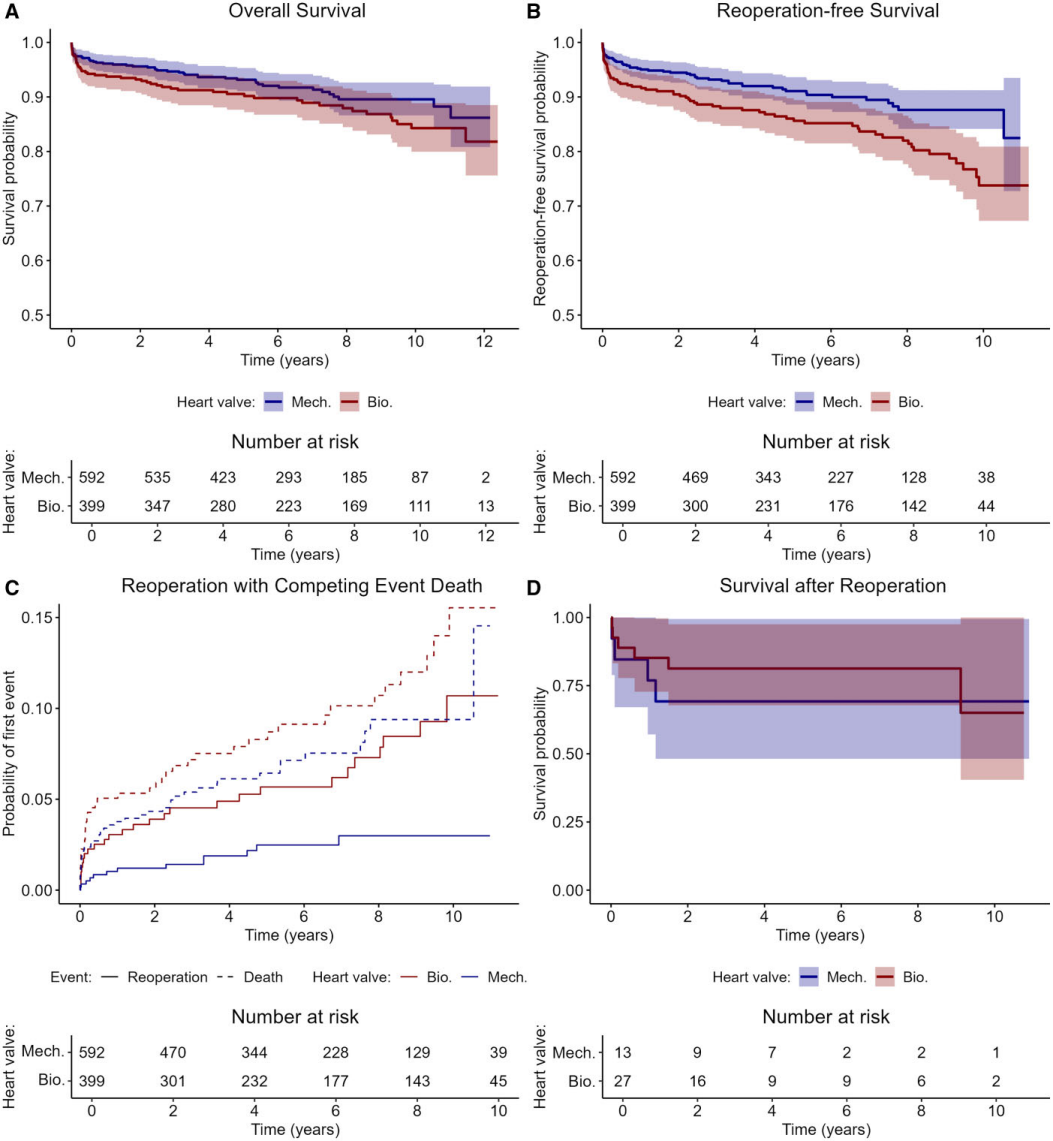
Kaplan–Meier curves for overall survival (**A**), reoperation-free survival (**B**) and survival after reoperation (**D**) as well as cumulative incidence curves for reoperation with competing event death (**C**).

The median follow-up time was 6.2 years (IQR: 3.5–9.5) up to a maximum of 12.4 years. The median follow-up was 6.8 years (IQR: 3.3–10.3) in the sB-AVR group and 6.0 years (IQR: 3.6–8.7) in the sM-AVR group. Ten-year overall survival rates were estimated with 0.90 (CL: 0.87–0.93) for sM-AVR and 0.84 (CL: 0.80–0.89) for the sB-AVR group. Although reduced survival for bioprostheses was observed, statistical analysis did not show a significant association between the type of sAVR and survival [HR = 1.216 (CL: 0.806–1.834), *P* = 0.352]. In contrast, age, diagnosis of HF, valvular and inflammatory cardiopathies as well as cardiomyopathies and pulmonary diseases were significantly associated with reduced survival (Table 1 and Fig. 2A). However, reoperation-free survival was significantly reduced in patients receiving bioprostheses [HR = 1.560 (95% CI: 1.076–2.262), *P* = 0.019] with 54 events (reoperation or death) in the sM-AVR group and 67 events in the bioprosthesis group. Estimated ten-year reoperation-free survival rates were 0.88 (CL: 0.84–0.91) for sM-AVR and 0.74 (CL: 0.67–0.81) for the sB-AVR group (Table 1 and Fig. 2B).

**Table 1:** Hazard ratios with corresponding 95% confidence interval of survival and reoperation-free survival (Cox regression) as well as reoperation (competing risk analysis)

	All-cause death		Death or reoperation		Reoperation	
	HR (95% CI)	*P*-Value	HR (95% CI)	*P*-Value	HR (95% CI)	*P*-Value
Heart valve (bio versus mech)	1.216 (0.806–1.834)	0.352	1.560 (1.076–2.262)	0.019	2.770 (1.402–5.472)	0.003
Age (per 1-year increase)	1.034 (1.004–1.066)	0.027	1.015 (0.990–1.041)	0.245	0.976 (0.936–1.018)	0.250
Sex (female versus male)	1.051 (0.658–1.677)	0.835	1.036 (0.684–1.569)	0.868	1.169 (0.563–2.428)	0.680
Heart failure before operation	2.201 (1.183–4.093)	0.013	1.387 (0.742–2.594)	0.305	0.298 (0.032–2.771)	0.290
Myocardial infarction before OP	2.353 (0.680–8.150)	0.177	2.816 (0.946–8.379)	0.063	4.905 (0.801–30.025)	0.085
Embolic stroke or ICH before OP	2.466 (0.976–6.235)	0.056	2.062 (0.846–5.022)	0.111	1.157 (0.121–11.075)	0.900
Diabetes mellitus before OP	1.768 (0.796–3.926)	0.162	1.579 (0.715–3.487)	0.259	NA	NA
Adiposity before OP	1.503 (0.713–3.167)	0.284	1.438 (0.688–3.005)	0.334	0.558 (0.069–4.521)	0.580
Hyperlipidaemia before OP	0.821 (0.405–1.664)	0.584	0.953 (0.503–1.804)	0.882	0.960 (0.287–3.212)	0.950
Hyperuricaemia/gout before OP	0.672 (0.140–3.221)	0.619	NA	NA	NA	NA
Cardiomyopathies before OP	2.044 (1.060–3.943)	0.033	2.048 (1.100–3.814)	0.024	1.669 (0.482–5.776)	0.420
Valvular cardiopathies before OP	0.498 (0.312–0.795)	0.003	0.603 (0.398–0.914)	0.017	0.961 (0.489–1.887)	0.910
Hypertensive cardiopathies before OP	1.325 (0.771–2.275)	0.308	1.014 (0.612–1.682)	0.956	1.079 (0.449–2.595)	0.860
Inflammatory cardiopathies before OP	1.940 (1.055–3.568)	0.033	1.771 (0.997–3.145)	0.051	1.182 (0.311–4.486)	0.810
Rhythmogenic cardiopathies before OP	0.907 (0.354–2.323)	0.839	1.364 (0.638–2.915)	0.423	2.616 (0.785–8.719)	0.120
Ischaemic heart disease before OP	1.037 (0.610–1.763)	0.894	0.964 (0.582–1.597)	0.887	0.732 (0.255–2.101)	0.560
Atherosclerosis before OP	3.652 (1.002–13.316)	0.050	3.634 (1.169–11.298)	0.026	1.716 (0.158–18.678)	0.660
Pulmonary diseases before OP	3.778 (1.527–9.349)	0.004	3.199 (1.313–7.792)	0.010	NA	NA
Kidney disease before OP	1.676 (0.838–3.350)	0.144	1.436 (0.733–2.813)	0.291	0.897 (0.173–4.650)	0.900

Covariables with NA entries were not included in the model, since not enough data were available to compute the estimators and *P*-values. Hazard ratios for the valve type are presented as bioprostheses versus mechanical prosthesis; thus, a hazard ratio larger than 1 indicates an increased probability of the corresponding event in the bioprostheses group (and vice versa).

CI: confidence interval; HR: hazard ratio; ICH: intracerebral haemorrhage.

In more detail, the multivariable competing risk model showed a significantly increased risk of reoperation in patients with bioprostheses [HR = 2.770 (CL: 1.402–5.472), *p* = 0.003] with none of the other parameters significantly associated (Table 1 and Fig. 2C). Ten-year reoperation probabilities were estimated to be 0.03 (CL: 0.02–0.05) for sM-AVR as compared to 0.11 (CL: 0.07–0.16) for sB-AVR. In total, 40 patients needed another surgical intervention, of those 13 patients had mechanical AVR and 27 patients bioprostheses as index operation.

**Figure 3:**
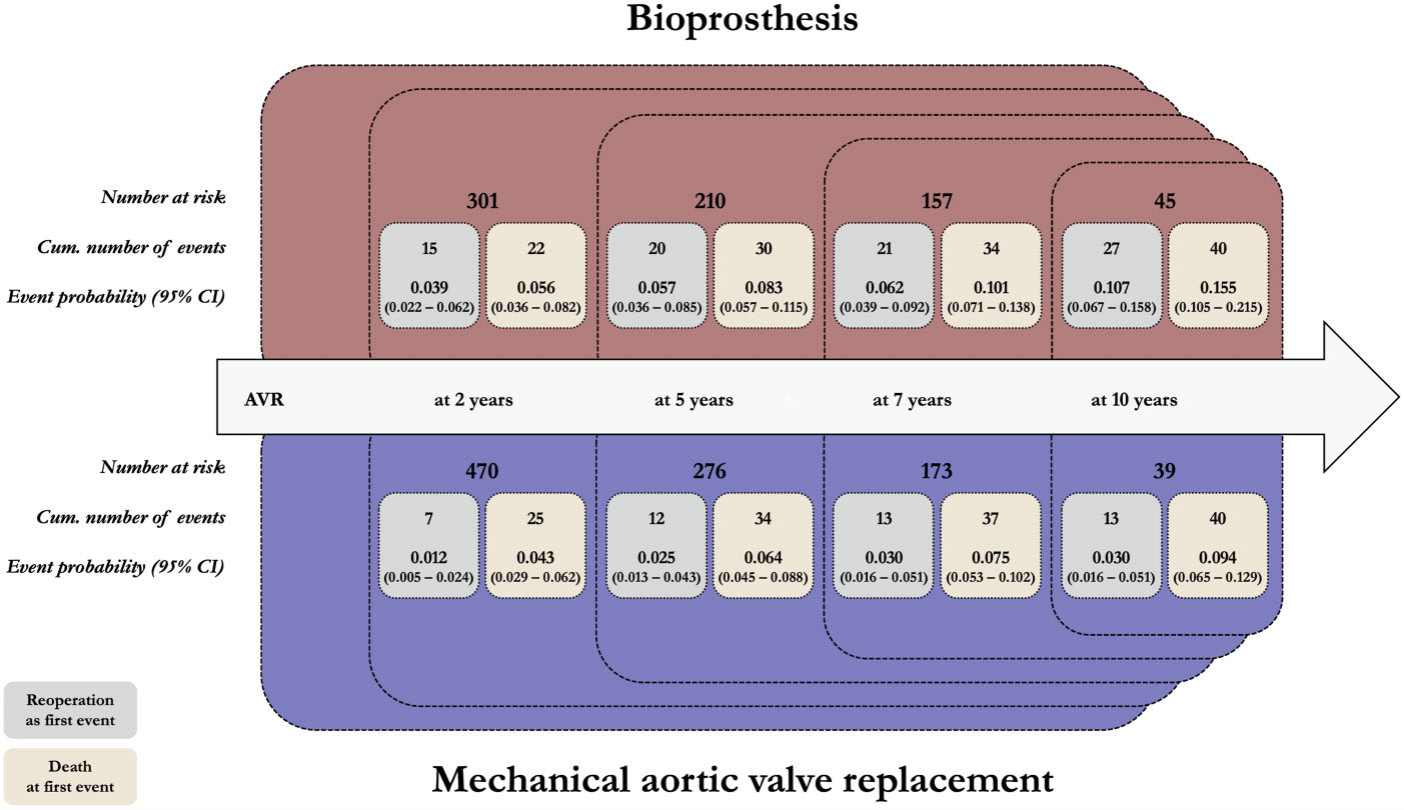
Number at risk, cumulative number of events and event probability with 95% confidence interval for outcome death or reoperation as the first event per group. AVR: aortic valve replacement.

Detailed event probability and cumulative number of events of both reoperation and death (as first event) in both groups at 2, 5, 7 and 10 years after AVR are given in Fig. 3. At all timepoints, the event probability of reoperation or death as first event is higher in patients receiving bioprostheses.

Survival after reoperation was observed to be impaired in both groups. After reoperation, an overall 2-year mortality probability of 0.23 (CL: 0.08–0.35) and a 10-year mortality probability of 0.34 (CL: 0.06–0.53) was estimated.

In more detail, a mortality rate of 0.31 (CL: 0.01–0.52) was found for sM-AVR and 0.35 (CL: 0.0–0.60) for sB-AVR as index operation at 10 years after reoperation (without statistical significance). Note that the survival probabilities after reoperation are based on only a small number of events. However, it has to be mentioned that even though we did observe similar mid-term survival in both groups after reoperation, the probability of reoperation was significantly higher in the sB-AVR group. A detailed table of types of reoperations can be found in Supplementary Material, Table S9. Kaplan–Meier curves for survival after reoperation separately for the index-op groups are given in Fig. 2D.

Results of further evaluated outcomes revealed that the type of AVR was not significantly associated with post-operative diagnosed myocardial infarction [HR = 0.805 (CL: 0.260–2.498), *p* = 0.71], embolic stroke or ICB [HR = 1.061 (CL: 0.562–2.004), *p* = 0.86], HF [HR = 0.861 (CL: 0.537–1.380), *p* = 0.53], bleeding other than ICB [HR: 0.873 (CL: 0.436–1.747), *p* = 0.70] or MACE [HR = 1.164 (CL: 0.867–1.562), *p* = 0.31]. For details, see Supplementary Material, Tables S10 and S11 and Supplementary Material, Figs S1 and S2. However, due to the small number of events, the results may be underpowered and due to the given study follow-up, events that may occur in a longer follow-up cannot be taken into account.

Estimated 10-year event probabilities in the sM-AVR group were 0.02 (CL; 0.01–0.04) for myocardial infarction; 0.06 (CL: 0.04–0.09) for embolic stroke or ICH; 0.10 (CL: 0.07–0.14) for HF, 0.05 (CL: 0.03–0.07) for bleeding other than ICH and 0.25 (CL: 0.19–0.30) for MACE. In the sB-AVR group, 10-year event rates were 0.02 (CL: 0.01–0.05) for myocardial infarction; 0.07 (CL: 0.04–0.10) for embolic stroke or ICH; 0.09 (CL: 0.06–0.13] for HF; 0.04 (CL: 0.02–0.07) for bleeding other than ICH; and 0.34 (CL: 0.27–0.41) for MACE. Detailed numbers for 2-, 5-, 7- and 10-year event probabilities can be found in Supplementary Material, Table S12.

## DISCUSSION

We found that our cohort of patients who underwent sM-AVR and were <50 years of age, did not show a significant difference in overall survival as compared to sB-AVR but had a significantly higher reoperation-free survival probability. The implantation of sB-AVR was associated with a significantly increased reoperation risk. Estimated 10-year mortality rate after reoperation was 34%, over both groups.

Implantation numbers show that sB-AVR was used regularly in Austria over the last years. Contrary to the recommendations of the ESC, a total of 40% of aortic valve recipients aged <50 years received sB-AVR. This reality contradicts both ESC and AHA guidelines [3, 9]. Most recently, the 2020 guidelines from AHA/ACC lowered these recommended age limits to >50 years for sB-AVR, arguing that these bioscaffolds have been demonstrated to improve haemodynamic status, have a lower risk of thromboembolic complications and not require lifelong anticoagulant therapy [3]. In our study, we evaluated several outcomes, including myocardial infarction, HF, embolic stroke or ICH and bleeding events other than ICH. In none of these outcomes, a significant difference between groups was observed; however, due to the small number of events, these results may be underpowered.

Many clinical studies have critically discussed the assumption that thromboembolic complications and haemodynamic status are better in patients who received sB-AVR versus sM-AVR [5, 6]. In relation to haemodynamics, it is accepted that the implantation of a bioprosthesis leads to an age-dependent development of subclinical structural valve degeneration (SVD) resulting in increased cardiac strain to the subvalvular myocardium [7]. This observation makes sense, given that recent studies described that >40% of all patients aged <65 years who received a bioprosthesis developed SVD [10, 11]. Only 2 reports have shown that patients undergoing sB-AVR may develop earlier HF as compared with patients receiving sM-AVR [6, 12]. This clinical finding supports the above notion that SVD does augment preload and facilitates the development of HF.

The early morphologic correlate for SVD was demonstrated recently. Radiologic investigations showed that the implantation of sB-AVR and TAVR is associated with an increased incidence of hypoattenuated leaflet thickening and reduced leaflet motion early after sB-AVR [13]. These results suggest that an early and prompt thrombotic and immunological process directed against the biological scaffold is initiated after surgical/catheter valve intervention. Our group and others have shown that the implantation of bovine/porcine valves elicits an alpha-Gal-specific immune response that is associated with clinically proven SVD [14]. The role of this humoral response against glycans in bioprosthetic heart valve deterioration was most prominently confirmed in a *Nature Medicine* contribution [15]. This consortium evidenced that anti-alpha-Gal, anti-Neu5Gc IgG expression as well as increased complement deposition at the bioprosthesis is initiated after a biological prosthesis is implanted. These findings agree with the findings of Veraar *et al.* [16]. They have shown that TAVR technology elicited a mid-term-specific humoral immune response that includes alpha-Gal antibodies, the complement system (C3a), citrullinated H3 levels and inflammatory soluble suppression of tumorigenesis-2. The academic consensus is that an age-dependent humoral and cellular immune response is responsible for SVD [17–19].

### ‘The physician’s advice is the patient’s choice’

By looking at the numbers of bioprostheses implanted in Austria in the years 2010–2020 sB-AVRs were used regularly contrary to the recommendations of the ESC, and it seems that patient’s choice of sB-AVR is superseding sM-AVR despite increased mortality in patients younger than 65 (observation period 2010–2018) [6]. The cardiologist and the cardiac surgeon know their patients best and will give their insights about the caveats and promises of available surgical interventions. Only the implantation of sM-AV will make lifelong Vit K-antagonist treatment mandatory. For example, women in childbearing age may therefore decide more often for sB-AVR. Indeed, in our cohort, we observed that slightly more women under the age of 40 decided for an sB-AVR (53.76%), however, the reason for their choice cannot be evaluated from our data and would be pure speculation. Note that Ross procedure has recently been discussed as an alternative to sM-AVR with similar survival in this patient group [20]. However, there seems to be no free lunch for the decision to sB-AVR and the widespread opinion that patients remaining lifespan will continue without major complications should be critically discussed [21].

A further explanation for patients opting for biological scaffolds may be that medical care and its availability after surgery can become difficult due to a potential larger risk of thromboembolic complications and requirement of lifelong anticoagulant therapy for sM-AVR. Such reasons are low availability of medical care in their vicinity, lack of medical insurance coverage, fear of losing medical insurance, an expensive and unaffordable health care system, and living in a low- and middle-income country with all its constraints. However, this may not be true in Austria, a European social welfare state with a public health care system, easily accessible for the Austrian population. It should be stated at this point that some lead publications that claim equality of sB-AVR and sM-AVR were registry studies from the USA [22, 23].

### Prominent representation creates a mental picture: did academia ask the right questions?

Summarizing from the historical perspective: multiple academic institutions have published short to mid-term results in patients aged <65 years that suggest that sB-AVR fares better than sM-AVR [4, 24]. We found only 1 contribution that evaluated sB-AVR in a comparison to sM-AVR in a group of patients with a mean age 50 years. Bruscky *et al.* [25] were the first to show that sM-AVR resulted in a significantly better outcome when looking at a combined end point of survival and reoperation. Our results support this observation in a larger cohort and we furthermore extend our results by reporting mortality probabilities after reoperation, which was estimated to be 34% after 10 years over both groups.

In this context, one may expect that 10–15 years after their index operation, patients aged <50 years will face a projected 25% reoperation risk in the sB-AVR group as compared with 10% in the sM-AVR group [23].

Interpreting the literature from a different perspective, we may ask why patient-relevant information, such as reoperation as well as combined freedom from reoperation and survival, were not discussed in more detail in former publications [4].

### Outlook

Several promising techniques have been reported to potentially increase the longevity of biological valves (BVs) used in cardiac surgery. In 2013, pretreatment of BVs was reported to effectively remove alpha-Gal epitopes from both bovine and porcine tissues [26, 27]. In addition to preservation techniques, there is growing interest in developing Gal-free BV from Gal-knockout pigs to engineer biological heart valves out of porcine pericardial leaflets, with excellent haemodynamics, long-term durability and no thrombogenicity in a sheep model [28]. Of interest are reports indicating that the generation of a bovine knockout for alpha-Gal and N-glycolylneuraminic acids, the major xenogeneic antigens, is feasible [29].

### Limitations

Even though our study provides real-world data, the registry from the insurance carriers covering 98% of Austrian citizens, the study design still has some limitations.

First, our data were obtained retrospectively and do not meet the criteria for a prospective randomized study with controlled treatment allocation. Second, even though we obtained data on diagnoses and medication, we cannot eliminate the possibility that sM-AVR was performed more often in primarily healthier patients. However, both groups show a similar distribution of previous diseases and relevant factors were included as confounders in multivariable models. However, to avoid overfitting or multicollinearity, we restricted the number of covariates to be not too large and covariates may therefore be heterogeneous. Third, although many patients were available, data on survival, reoperation and other secondary outcomes must be examined with caution, as the event rate may still be low and thus possible differences might not be detected. Furthermore, due to the study follow-up of maximal 12 years, events that may occur in a longer follow-up cannot be taken into account. Nevertheless, AVR in patients <50 years is per definition infrequently performed, and this is to our knowledge the largest study investigating bioprostheses and mechanical aortic valve prostheses in young (<50 years) patients. Fourth, since we analysed administrative data, data is derived from billing information and discharge coding. We therefore rely on correct nationwide coding of diseases and events for a reliable evaluation of our data. This is something that cannot be retrospectively verified or corrected and might result in biased results and conclusions may lead to discrepancies compared to prospective clinical databases [30]. Furthermore, we were not able to evaluate medical causes of death or reoperation in detail from the given database. The exclusion of patients with causes of death or reoperation unrelated to the index surgery was therefore not possible. However, all major outcome parameters analysed for this study are events that require hospital care and may therefore be reported precisely to the Austrian Health Insurance Carriers for billing purposes.

## CONCLUSIONS

From the epistemological standpoint we believe that our data give a detailed insight in mid-term AVR outcomes over the last decade. We present results of a national population-based cohort. Follow-up in our cohort is nearly 100% and follows preoperative international disease codes and documents prescribed medications prior to the index operation. Moreover, MEL codes correspond to the reimbursement of all Austrian cardiac surgery departments that are funded by 1 insurance carrier.

Regarding AVR in patients aged <50 years, we found that patients had a similar morbidity before the index surgery. Although no significant difference in overall survival between sB-AVR and sM-AVR was observed, our data analyses found that a significantly larger probability of reoperation was found for sB-AVR (11%) as compared to sM-AVR (3%) within 10 years. Ten-year mortality after reoperation was estimated to be 34% over both groups. This leads to the conclusion that the decision for sB-AVR or sM-AVR should be critically discussed in young patients. sB-AVR may not make lifelong VitK-antagonist treatment mandatory; however, this may come to the cost of a larger reoperation probability.

## Supplementary Material

ezad308_Supplementary_Data

## Data Availability

Pseudonymized participant data will be made available upon qualified request starting with publication. Approval of a proposal and a signed data access agreement are mandatory.

## ABBREVIATIONS

AHA/ACC: American Heart Association/American College of Cardiology
AVR: Aortic valve replacement
BVs: Biological valves
ESC: European Society of Cardiology
HF: Heart failure
HR: Hazard ratio
ICD-10: International Statistical Classification of Diseases and Related Health Problems in its 10th revision
ICH: Intracerebral haemorrhage
IQR: Interquartile range
MACE: Major adverse cardiac event
sAVR: Surgical aortic valve replacement
sB-AVR: Surgical/biological aortic valve replacement
sM-AVR: Surgical/mechanical aortic valve replacement
SVD: Structural valve degeneration
TAVR: Transcatheter aortic valve replacement
