# Supplementary material for: Revisiting aortic valve prosthesis choice in patients younger than 50 years: 10 years results of the AUTHEARTVISIT study
Source: Eur J Cardiothorac Surg. 2023 Sep 26;65(1):ezad308. doi: 10.1093/ejcts/ezad308 (PMC10761203; doi:10.1093/ejcts/ezad308)
Supplement: ezad308_Supplementary_Data [file ezad308_supplementary_data.zip › Supplement_Revision_R3_170823.docx]

**Supplement to**

**Revisiting prosthesis choice in patients younger than 50 years: 10 years results of the AUTHEARTVISIT Study**

1. **General Information on Coding**

For each patient, billing information (based on MEL-Codes from the Austrian Insurance Carriers) and diagnoses (based on ICD-10-codes) were available from 1 year before index-op to study closure.

To evaluate the diagnoses, the International Statistical Classification of diseases and Related Health Problems in its 10th revision (ICD-10 version of 2019) was used, which is available on:

[**https://icd.who.int/browse10/2019/en**](https://icd.who.int/browse10/2019/en)

The corresponding German version is available on:

[**https://www.dimdi.de/static/de/klassifikationen/icd/icd-10-who/kode-suche/htmlamtl2019/**](https://www.dimdi.de/static/de/klassifikationen/icd/icd-10-who/kode-suche/htmlamtl2019/)

The following Table shows the MEL codes used for the inclusion and exclusion criteria as well as for the definition of outcomes.

| **MEL Code** | **Description** |
| --- | --- |
| DB020 | percutaneous implantation of a pulmonary valve |
| DB025 | Aortic valve replacement – catheter directed, transapikal, TAVR |
| DB026 | Aortic valve replacement – catheter directed, transvalvular, TAVR |
| DB030 | Reconstruction of the aortic valve |
| DB040 | Reconstruction of the mitral valve |
| DB050 | Reconstruction of the tricuspid valve |
| DB055 | Reconstruction of the pulmonary valve |
| DB060 | Replacement of aortic valve with pulmonary autograft |
| DB070 | Replacement of aortic valve with stentless valve |
| DB080 | Replacement of aortic valve with stented valve |
| DB082 | Replacement of aortic valve with artificial mechanical valve |
| DB090 | Replacement of mitral valve with stentless valve |
| DB100 | Replacement of mitral valve with stented valve |
| DB102 | Replacement of mitral valve with artificial mechanical valve |
| DB110 | Replacement of tricuspid valve with stentless valve |
| DB120 | Replacement of tricuspid valve with stented valve |
| DB122 | Replacement of tricuspid valve with artificial mechanical valve |
| DB130 | Replacement of pulmonary valve with stentless valve |
| DB140 | Replacement of pulmonary valve with stented valve |
| DB142 | Replacement of pulmonary valve with artificial mechanical valve |
| DB021 | Aortic valve replacement – percutaneous, interventional, TAVR |
| XN010 | Aortic valve replacement – percutaneous, interventional, TAVR |

**Supplementary Table 1:** MEL Codes used for Inclusion and Exclusion Criteria as well as for Definitions of outcomes and confounders.

1. **Inclusion and Exclusion Criteria**

Clinical and operative data of all patients registered in the Austrian Health Care System who underwent sAVR (either using a mechanical aortic valve replacement “sM-AVR” - MEL Code DB082 - or a bioprosthetic aortic valve replacement “sB-AVR” - MEL Codes DB060, DB070, and DB080) in Austria in the years 2010 - 2020 at an age below 50 years were obtained. For description of MEL Codes see supplementary Table 1

Patients receiving transcatheter aortic valve replacement (TAVR: MEL-Codes DB025, DB026, DB021 or XN010, see supplementary Table 1) as index op were not included in the sample.

Patients aged <18 years were excluded from the data.

Patients with concomitant heart surgery were excluded from the data, i.e. patients with at least one valve surgery additionally to the index OP (sM-AVR or sB-AVR) at index op date (used MEL codes listed in supplementary Table 1).

Furthermore, patients receiving a coronary artery stent (MEL Code DD050 “implantation of a stent in the coronary artery“ or DD060 “implantation of a drug eluting stent in a coronary artery”) within 4 months prior to aortic valve replacement were excluded from this analysis. We selected a period of four months previous to the surgery for exclusion of patients with percutaneous coronary intervention to guarantee selection of patients with pure AVR procedures.

1. **Detailed Description of Statistical Methods**

Continuous data are presented descriptively as median with interquartile range (IQR), while categorical data are shown as counts and percentages. Categorical variables were compared between both groups using chi-squared test.

For survival, Cox regression and Kaplan-Meier curves were used to evaluate the benefit of one valve type on overall survival (sM-AVR vs. sB-AVR). For multivariable analysis, the following covariables were additionally included as possible confounders to the statistical model: age (in years), sex (female vs. male), heart failure, myocardial infarction, embolic stroke or intracerebral hemorrhage (ICH), diabetes mellitus, adiposity, hyperlipidemia, hyperuricemia/gout, valvular, inflammatory, rhythmogenic or hypertensive cardiopathies, cardiomyopathies, ischemic heart disease, atherosclerosis, pulmonary diseases and kidney diseases prior to operation. These comorbidities were defined using ICD-Codes available for each patient up to one year before index-op. Data available before index-op were scanned for each patient based on ICD-Codes, categorized for the different co-morbidities. If at least one time in the year before index-op an ICD-Code for a co-morbidity was observed as main or secondary diagnoses, the patient was assumed to suffer from the comorbidity.

The combined endpoint for “reoperation or death “was analysed using a multivariable Cox regression model including the same covariables as for the survival endpoint. To investigate reoperation in more detail, competing risk regression for the endpoint reoperation with competing event death was performed. Same covariables as for the survival endpoint were included in the model.

Proportional hazard assumption was evaluated using Schönfeld residuals, multicollinearity was evaluated using variance inflation factors.

The further secondary endpoints “heart failure”, “myocardial infarction”, “embolic stroke or ICH” and “bleeding other than ICH” were evaluated by competing risk analysis using death as competing event. Same covariables as for the survival endpoint were included in the model.

The secondary endpoint “major adverse cardiac events” (MACE) was analysed using Cox regression models. Same covariables as for the survival endpoint were included in the model.

For all analyses, hazard ratios (HR) and survival probabilities, corresponding 95% confidence intervals are presented. Note that for the secondary endpoints MACE and HF, patients with a diagnosed HF before the surgery were excluded to focus on newly diagnosed events after surgery. HR are presented as sB-AVR vs. sM-AVR, thus a HR larger than 1 indicates an increased probability of the corresponding event in the bioprostheses group (and vice versa).

Statistical analyses were carried out and graphs generated in R (version 4.1.3) using the following packages: survival (version 3.2.13), survminer (version 0.4.9) and cmprsk (2.2.11). All tests were performed at a two-sided significance level of 0.05.

1. **Definition of Outcomes**

For each patient, billing information (based on MEL-Codes) and diagnoses (based on ICD-10-codes) were available from 1 year before index-op to study closure. Furthermore, death dates were available until the time of study closure. To evaluate the different outcomes for each patient, data was scanned from index-op to study closure for the corresponding codes, shown in the following tables.

| **Outcome** | **Definition** |
| --- | --- |
| Death | All-cause death based on death date |
| Reoperation | Based on billing information (MEL-Code): first event after index-op with MEL code defined as in Supplementary Table 1 |
| Death or Reoperation | Combined endpoint: first event after index-op with MEL code defined as in Supplementary Table 1 or death based on death date |
| Myocardial Infarction | Based on ICD-Codes: first event after index-op with ICD-code defined as in Supplementary Table 3 |
| Heart Failure | Based on ICD-Codes: first event after index-op with ICD-code defined as in Supplementary Table 3 |
| Embolic Stroke or intracerebral hemorrhage (ICH) | Based on ICD-Codes: first event after index-op with ICD-code defined as in Supplementary Table 3 |
| Bleeding other than ICH | Based on ICD-Codes: first event after index-op with ICD-code defined as in Supplementary Table 3 |
| MACE | Combined endpoint: first event after index-op with ICD-codes for Myocardial Infarction, Heart Failure, Embolic Stroke or ICH, Reoperation or death |

**Supplementary Table 2:** Definition of outcomes

| **Outcome** | **ICD-10-Codes** |
| --- | --- |
| Myocardial Infarction | I21.0, I21.1, I21.2, I21.3, I21.4, I21.9 |
| Heart Failure | I11.0, I13.0, I13.2, I50.0, I50.1, I50.9, I50.11, I50.12, I50.13, I50.14, I50.19 |
| Embolic Stroke or intracerebral hemorrhage (ICH) | I63.0, I63.1, I63.2, I63.3, I63.4, I63.5, I63.6, I63.8, I63.9, G45.9, G45.0, G45.1, G45.2, G45.3, G45.4, G45.8, I61.0, I61.1, I61.2, I61.3, I61.4, I61.5, I61.6, I61.8, I61.9, I64 |
| Bleeding other than ICH | I60.0, I60.1, I60.2, I60.3, I60.4, I60.5, I60.6, I60.7, I60.8, I60.9, I85.0, I98.2, I98.3, K25.0, K25.1, K25.2, K25.3, K25.4, K25.5, K25.6, K25.7, K25.9, K26.0, K26.1, K26.2, K26.3, K26.4, K26.5, K26.6, K26.7, K26.9, K27.0, K27.1, K27.2, K27.3, K27.4, K27.5, K27.6, K27.7, K27.9, K28.0, K28.1, K28.2, K28.3, K28.4, K28.5, K28.6, K28.7, K28.9, K29.0, K29.1, K29.2, K29.3, K29.4, K29.5, K29.6, K92.2, N42.1, R04.1, R04.8, R04.9, R58, S06.4, T81.0 |

**Supplementary Table 3:** Definitions for Outcomes based on ICD-10-Codes

1. **Definitions of Confounders/Comorbidities**

The index-op group was defined using billing information (based on MEL-Codes):

| **MEL Code** | **Description** | **Group** |
| --- | --- | --- |
| DB060 | Replacement of aortic valve with pulmonary autograft | sB-AVR |
| DB070 | Replacement of aortic valve with stentless valve | sB-AVR |
| DB080 | Replacement of aortic valve with stented valve | sB-AVR |
| DB082 | Replacement of aortic valve with artificial mechanical valve | sM-AVR |

**Supplementary Table 4:** Coding for grouping variable, sB-AVR: Bioprostheses; sM-AVR: mechanical aortic valve replacement

Comorbidities were defined using ICD-10-Codes available for each patient up to one year before index-op. Data available one year before index-op were scanned for each patient based on the following ICD-10-Codes, categorized for different comorbidities (Supplementary Table 5). If at least one time in the year before index-op an ICD-10-Code for a comorbidity was observed as main or secondary diagnoses, the patient was assumed to suffer from this comorbidity.

| **Comorbidities before OP** | **ICD-10-Codes** |
| --- | --- |
| Diabetes mellitus | E10.0, E10.1, E10.2, E10.3, E10.4, E10.5, E10.6, E10.7, E10.8, E10.9, E11.0, E11.1, E11.2, E11.3, E11.4, E11.5, E11.6, E11.7, E11.8, E11.9, E12.0, E12.1, E12.2, E12.3, E12.4, E12.5, E12.6, E12.7, E12.8, E12.9, E13.0, E13.1, E13.2, E13.3, E13.4, E13.5, E13.6, E13.7, E13.8, E13.9, E14.0, E14.1, E14.2, E14.3, E14.4, E14.5, E14.6, E14.7, E14.8, E14.9 |
| Adiposity | E65, E66.0, E66.1, E66.2, E66.8, E66.9 |
| Hyperlipidemia | E78.0, E78.1, E78.2, E78.3, E78.4, E78.5, E78.6, E78.8, E78.9 |
| Hyperuricemia/Gout | E79.0, E79.8, M10.0, M10.00, M10.01, M10.02, M10.03, M10.04, M10.05, M10.06, M10.07, M10.08, M10.09 |
| Cardiomyopathies | I42.0, I42.1, I42.2, I42.5, I42.6, I42.7, I42.8, I42.9, I43.0, I43.1, I43.2, I43.8, I51.8 |
| Valvular cardiopathies | I05.0, I05.1, I05.2, I05.8, I05.9, I06.0, I06.1, I06.2, I06.8, I06.9, I07.0, I07.1, I07.2, I07.8, I07.9, I08.0, I08.1, I08.2, I08.3, I08.8, I08.9, I34.0, I34.1, I34.2, I34.8, I34.9, I35.0, I35.1, I35.2, I35.8, I35.9, I36.0, I36.1, I36.2, I36.8, I36.9, I37.0, I37.1, I37.2, I37.8, I37.9, I39.0, I39.1, I39.2, I39.3, I39.4, I42.4, Q21.0, Q21.1, Q21.3, Q22.1, Q22.2, Q22.3, Q22.4, Q22.5, Q22.8, Q22.9, Q23.0, Q23.1, Q23.2, Q23.3, Q23.8, Q23.9, Q24.3, Q24.4, Q25.3 |
| Hypertensive cardiopathies | I10, I11.9, I12.0, I12.9, I13.1, I13.9, I15.0, I15.1, I15.2, I15.8, I15.9, I27.0, I27.2, I27.8, I27.9 |
| Inflammatory cardiopathies | I01.0, I01.1, I01.2, I01.8, I01.9, I02.0, I02.9, I09.0, I09.1, I09.2, I09.8, I09.9, I30.0, I30.1, I30.8, I30.9, I31.0, I31.8, I31.9, I32.0, I32.1, I32.8, I33.0, I33.9, I38, I39.8, I40.0, I40.1, I40.8, I40.9, I41.0, I41.1, I41.2, I41.8, I42.3, I51.4 |
| Rhythmogenic cardiopathies | I44.0, I44.1, I44.2, I44.3, I45.1, I45.2, I45.4, I45.6, I45.9, I46.0, I46.1, I46.9, I47.0, I47.1, I47.2, I47.9, I48.0, I48.1, I48.2, I48.3, I48.4, I48.9, I49.0, I49.1, I49.2, I49.3, I49.4, I49.5, I49.8, I49.9 |
| Atheriosclerosis | I69.8, I70.0, I70.1, I70.2, I70.8, I70.9 |
| Pulmunary Disease | J43.1, J43.2, J43.8, J43.9, J44.00, J44.01, J44.02, J44.03, J44.09, J44.10, J44.11, J44.12, J44.13, J44.19, J44.80, J44.81, J44.82, J44.83, J44.89, J44.90, J44.91, J44.92, J44.93, J44.99, J45.0, J45.1, J45.8, J45.9 |
| Kidney disease | N00,0, N00.1, N00.2, N00.3, N00.4, N00.5, N00.6, N00.7, N00.8, N00.9, N01.0, N01.1, N01.2, N01.3, N01.4, N01.5, N01.6, N01.7, N01.8, N01.9, N02.0, N02.1, N02.2, N02.3, N02.4, N02.5, N02.6, N02.7, N02.8, N02.9, N03.0, N03.1, N03.2, N03.3, N03.4, N03.5, N03.6, N03.7, N03.8, N03.9, N04.0, N04.1, N04.2, N04.3, N04.4, N04.5, N04.6, N04.7, N04.8, N04.9, N05.0, N05.1, N05.2, N05.3, N05.4, N05.5, N05.6, N05.7, N05.8, N05.9, N06.0, N06.1, N06.2, N06.3, N06.4, N06.5, N06.6, N06.7, N06.8, N06.9, N07.0, N07.1, N07.2, N07.3, N07.4, N07.5, N07.6, N07.7, N07.8, N07.9, N08.0, N08.1, N08.2, N08.3, N08.4, N08.5, N08.8, N10, N11.0, N11.1, N11.8, N11.9, N12, N13.0, N13.1, N13.2, N13.3, N13.4, N13.5, N13.6, N13.7, N13.8, N13.9, N14.0, N14.1, N14.2, N14.3, N14.4, N15.0, N15.1, N15.8, N15.9, N16.0, N16.1, N16.2, N16.3, N16.4, N16.5, N16.8, N17.0, N17.1, N17.2, N17.8, N17.9, N18.1, N18.2, N18.3, N18.4, N18.5, N18.9, N19, N20.0 |
| Ischemic heart diseases | I20.0, I20.1, I20.8, I20.9, I21.0, I21.1, I21.2, I21.3, I21.4, I21.9, I22.0, I22.1, I22.8, I22.9, I23.0, I23.1, I23.2, I23.3, I23.4, I23.5, I23.6, I23.8, I24.0, I24.1, I24.8, I24.9, I25.0, I25.1, I25.2, I25.3, I25.4, I25.5, I25.6, I25.8, I25.9 |
| Embolic Stroke or ICH | ICD-10-Codes as in Supplementary Table 3 |
| Heart failure | ICD-10-Codes as in Supplementary Table 3 |
| Myocardial Infarction | ICD-10-Codes as in Supplementary Table 3 |

**Supplementary Table 5:** ICD-10-codes for definition of comorbidities

1. **Different Surgery Types per Year**

|  | **sB-AVR** | | | **sM-AVR** |
| --- | --- | --- | --- | --- |
| Year | DB060 | DB070 | DB080 | DB082 |
| 2010 | 9 (8.91%) | 5 (4.95%) | 44 (43.56%) | 43 (42.57%) |
| 2011 | 5 (4.27%) | 5 (4.27%) | 56 (47.86%) | 51 (43.59%) |
| 2012 | 6 (6%) | 1 (1%) | 38 (38%) | 55 (55%) |
| 2013 | 3 (4.92%) | 1 (1.64%) | 10 (16.39%) | 47 (77.05%) |
| 2014 | 0 (0%) | 3 (3.85%) | 19 (24.36%) | 56 (71.79%) |
| 2015 | 0 (0%) | 1 (1.16%) | 36 (41.86%) | 49 (56.98%) |
| 2016 | 0 (0%) | 1 (1.12%) | 19 (21.35%) | 69 (77.53%) |
| 2017 | 0 (0%) | 3 (2.88%) | 30 (28.85%) | 71 (68.27%) |
| 2018 | 10 (10.53%) | 3 (3.16%) | 25 (26.32%) | 57 (60%) |
| 2019 | 12 (13.48%) | 3 (3.37%) | 17 (19.1%) | 57 (64.04%) |
| 2020 | 6 (8.45%) | 3 (4.23%) | 25 (35.21%) | 37 (52.11%) |

**Supplementary Table 6:** Distribution of Heart Valve Types within the study period. Percentages per year and absolute numbers of patients receiving mechanical aortic valve or bioprosthetic valve replacement per calendar year. sB-AVR: Bioprostheses; sM-AVR: mechanical aortic valve replacement

1. **Pre-existing medical diagnoses and medicaments**

|  | sM-AVR  (n=592) | sB-AVR  (n=399) | p-value |
| --- | --- | --- | --- |
| Heart failure | 37 (6.25%) | 35 (8.77%) | 0.17 |
| Myocardial Infarction | 6 (0.01%) | 9 (2.29%) | 0.19 |
| Embolic Stroke or ICH | 7 (1.18%) | 11 (2.76%) | 0.11 |
| Diabetes mellitus | 24 (4.1%) | 15 (3.8%) | 0.95 |
| Adiposity | 38 (6.4%) | 16 (4.0%) | 0.14 |
| Hyperlipidaemia | 54 (9.1%) | 42 (10.5%) | 0.53 |
| Hyperuricemia/gout | 5 (0.8%) | 4 (1.0%) | 1.00 |
| Cardiomyopathies | 41 (6.93%) | 23 (5.76%) | 0.55 |
| Valvular cardiopathies | 380 (62.5%) | 231 (57.89%) | 0.165 |
| Hypertensive cardiopathies | 111 (18.75%) | 73 (18.3%) | 0.923 |
| Inflammatory cardiopathies | 39 (6.59%) | 34 (8.52%) | 0.308 |
| Rhythmogenic cardiopathies | 30 (5.07%) | 17 (4.26%) | 0.664 |
| Ischemic cardiomyopathies | 112 (18.9%) | 75 (18.8%) | 1.0 |
| Atherosclerosis | 3 (0.5%) | 6 (1.5%) | 0.20 |
| Pulmonary diseases | 7 (1.2%) | 7 (1.8%) | 0.64 |
| Kidney diseases | 25 (4.2%) | 19 (4.8%) | 0.81 |
| Infectious diseases | 20 (3.4%) | 22 (5.5%) | 0.14 |
| Haematological disease | 0 (0.0%) | 1 (0.3%) | 0.84 |
| SIADH/PHA | 0 (0.0%) | 0 (0.0%) | NA |
| Vitamin D deficiency | 2 (0.3%) | 0 (0%) | 0.66 |
| Electrolyte disbalance | 2 (0.3%) | 2 (0.5%) | 1.00 |
| Amyloidosis | 0 (0.0%) | 0 (0.0%) | NA |
| Addiction | 12 (2.0%) | 15 (3.8%) | 0.15 |
| Smoking | 40 (6.8%) | 23 (5.8%) | 0.62 |
| Aortic disease | 63 (10.6%) | 26 (6.5%) | 0.03 |
| Dental/gingival diseases | 8 (1.4%) | 6 (1.5%) | 1.00 |
| Gastric/duodenal ulcers | 20 (3.4%) | 10 (2.5%) | 0.55 |
| Intestinal diseases | 3 (0.5%) | 7 (1.8%) | 0.11 |
| Liver diseases | 9 (1.5%) | 16 (4.0%) | 0.03 |
| Pancreatic diseases | 1 (0.1%) | 2 (0.5%) | 0.73 |
| Decubitus/pyoderma gangrenosum | 0 (0.0%) | 0 (0.0%) | NA |
| Rheumatic diseases | 7 (1.2%) | 5 (1.3%) | 1.0 |
| Transplantation (including complications) | 3 (0.5%) | 2 (0.5%) | 1.0 |

**Supplementary Table 7:** Pre-existing medical diagnoses at the time of the index operation. The numbers represent the number of patients with a diagnosis and the corresponding percentage of the total patients in the treatment group. The first 16 diagnoses (marked in grey) were included as covariables to the statistical models. sB-AVR: Bioprostheses; sM-AVR: mechanical aortic valve replacement

|  | sM-AVR  (n=592) | sB-AVR  (n=399) | p-value |
| --- | --- | --- | --- |
| Medication for hepatobiliary diseases | 0 (0.0%) | 1 (0.3%) | 0.84 |
| Intestinal anti-infectives | 3 (0.5%) | 5 (1.3%) | 0.36 |
| Intestinal antiphlogistics | 2 (0.3%) | 0 (0.0%) | 0.66 |
| Insulin | 10 (1.7%) | 7 (1.8%) | 1.00 |
| Non-insulin antidiabetics | 19 (3.2%) | 8 (2.0%) | 0.35 |
| Vitamin D | 9 (1.5%) | 11 (2.8%) | 0.26 |
| Electrolytes | 21 (3.6%) | 18 (4.5%) | 0.55 |
| Vitamin K antagonists | 21 (3.6%) | 11 (2.8%) | 0.61 |
| Heparins | 41 (6.9%) | 33 (8.3%) | 0.51 |
| Inhibitors of platelet aggregation | 26 (4.9%) | 21 (5.3%) | 0.63 |
| Direct oral anticoagulants | 5 (0.8%) | 2 (0.5%) | 0.81 |
| Iron therapy | 15 (2.5%) | 8 (2.0%) | 0.74 |
| Antianemic preparations | 4 (0.7%) | 4 (1.0%) | 0.84 |
| Glycosides | 1 (0.2%) | 0 (0.0%) | 1.00 |
| Anti-arrhythmic agents | 4 (0.7%) | 1 (0.3%) | 0.64 |
| Adrenergic and dopaminergic stimulants | 6 (1.0%) | 1 (0.3%) | 0.31 |
| Vasodilators | 9 (1.5%) | 7 (1.8%) | 0.98 |
| Antihypertensive drugs | 48 (8.1%) | 30 (7.5%) | 0.83 |
| New pulmonary hypertension therapy | 0 (0.0%) | 0 (0.0%) | NA |
| Loop diuretics | 25 (4.2%) | 16 (4.0%) | 1.00 |
| Aldosterone antagonist | 33 (5.6%) | 20 (5.0%) | 0.81 |
| Beta blockers | 88 (14.9%) | 49 (12.3%) | 0.29 |
| RAAS inhibitors | 134 (22.6%) | 84 (21.1%) | 0.61 |
| Lipid-lowering agents | 112 (18.9%) | 69 (17.3%) | 0.58 |
| Topical antibiotics | 8 (1.4%) | 9 (2.3%) | 0.41 |
| Dermal steroids | 21 (3.6%) | 19 (4.8%) | 0.43 |
| Parathyroid antagonists | 1 (0.2%) | 2 (0.5%) | 0.73 |
| Systemic antibiotics | 280 (47.3%) | 165 (41.4%) | 0.08 |
| Systemic antiviral drugs | 14 (2.4%) | 17 (4.3%) | 0.14 |
| Chemo-/Immunotherapies | 2 (0.3%) | 3 (0.8%) | 0.66 |
| Immunosuppressives | 16 (2.7%) | 11 (2.8%) | 1.00 |
| NSAIDs | 138 (23.3%) | 90 (22.6%) | 0.84 |
| Gout drugs | 9 (1.5%) | 6 (1.5%) | 1.00 |
| Bisphosphonates | 3 (0.5%) | 2 (0.5%) | 1.00 |
| Analgetics | 53 (9.0%) | 36 (9.0%) | 1.00 |
| Inhaled drugs for obstructive pulmonary diseases | 79 (13.3%) | 37 (9.3%) | 0.06 |
| Xanthines and leukotriene antagonists | 6 (1.0%) | 3 (0.8%) | 0.93 |

**Supplementary Table 8:** Pre-existing medication at the time of the index operation. The numbers represent the number of patients who had used a medication and the corresponding percentage of the patients in each group. RAAS, renin-angiotensin-aldosterone system; NSAIDs, non-steroidal anti-inflammatory drugs. sB-AVR: Bioprostheses; sM-AVR: mechanical aortic valve replacement

1. **Observed Reoperations**

| **Index-OP** | **sB-AVR** | **sM-AVR** |
| --- | --- | --- |
| Aortic valve replacement with stented valve | 6 | 1 |
| Combination: Aortic valve replacement with stented valve + Mitral valve replacement with stented valve | 1 | 0 |
| Aortic valve replacement with mechanical valve | 4 | 2 |
| Combination: Aortic valve replacement with mechanical valve + Mitral valve replacement with mechanical valve | 1 | 0 |
| Aortic valve replacement with pulmonary autograft | 1 | 0 |
| Aortic valve replacement without stented valve | 4 | 1 |
| Mitral valve replacement with stented valve | 3 | 0 |
| Mitral valve replacement with mechanical valve | 1 | 2 |
| Percutaneous pulmonary valve replacement | 1 | 0 |
| Reconstruction of aortic valve | 1 | 3 |
| Reconstruction of mitral valve | 1 | 0 |
| Combination: Aortic valve replacement with mechanical valve + Reconstruction of mitral valve | 0 | 1 |
| Reconstruction of mitral valve + Reconstruction of tricuspid valve | 2 | 0 |
| Combination: Aortic valve replacement with mechanical valve + Reconstruction of mitral valve + Reconstruction of tricuspid valve | 0 | 1 |
| Combination: Aortic valve replacement with stented valve + Mitral valve replacement with mechanical valve + Reconstruction of tricuspid valve | 0 | 1 |
| Combination: Aortic valve replacement with mechanical valve + Reconstruction of tricuspid valve + Mitral valve replacement with mechanical valve | 1 | 0 |
| Reconstruction of tricuspid valve + Mitral valve replacement with mechanical valve | 0 | 1 |

**Supplementary Table 9:** Types of observed reoperations, separately for index-op. sB-AVR: Bioprostheses; sM-AVR: mechanical aortic valve replacement

1. **Results for secondary outcomes**

9.1.) Outcomes: Myocardial infarction, Embolic stroke or ICH, Heart failure and Bleeding, other than ICH

|  | Outcome | | | | | | | |
| --- | --- | --- | --- | --- | --- | --- | --- | --- |
|  | **Myocardial Infarction** | | **Embolic Stroke or ICH** | | **Heart failure** | | **Bleeding, other than ICH** | |
|  | **HR (95% CI)** | **p-value** | **HR (95% CI)** | **p-value** | **HR (95% CI)** | **p-value** | **HR (95% CI)** | **p-value** |
| Heart valve  (sB-AVR) | 0.805  (0.260 - 2.498) | 0.71 | 1.061  (0.562 - 2.004) | 0.86 | 0.861  (0.537 - 1.380) | 0.53 | 0.873  (0.436 - 1.747) | 0.70 |
| Age (per one year increase) | 1.035  (0.956 - 1.121) | 0.39 | 1.004  (0.966 - 1.044) | 0.84 | 1.015  (0.985 - 1.046) | 0.34 | 0.994  (0.952 - 1.039) | 0.80 |
| Sex (female) | 0.343  (0.040 - 2.914) | 0.33 | 1.710  (0.900 - 3.246) | 0.10 | 0.969  (0.573 - 1.640) | 0.91 | 0.539  (0.206 - 1.410) | 0.21 |
| Heart failure* | 2.414  (0.497 - 11.730) | 0.27 | 0.547  (0.114 - 2.635) | 0.45 |  |  | 1.562  (0.490 - 4.976) | 0.45 |
| Myocardial infarction* | 4.142  (0.393 - 43.706) | 0.24 | 2.694  (0.600 - 12.092) | 0.20 | 0.728  (0.089 - 5.936) | 0.77 | 2.338  (0.252 - 21.688) | 0.45 |
| Embolic Stroke or ICH* | NA | NA | 2.281  (0.530 - 9.821) | 0.27 | 1.512  (0.411 - 5.565) | 0.53 | 1.552  (0.191 - 12.598) | 0.68 |
| Diabetes mellitus* | 1.250  (0.107 - 14.650) | 0.86 | 2.610  (0.769 - 8.855) | 0.12 | 2.561  (0.948 - 6.916) | 0.06 | 0.935  (0.187 - 4.689) | 0.94 |
| Adiposity* | NA | NA | 0.508  (0.116 - 2.223) | 0.37 | 1.482  (0.467 - 4.704) | 0.50 | 0.453  (0.082 - 2.514) | 0.37 |
| Hyperlipidemia* | 0.724  (0.106 - 4.935) | 0.74 | 2.702  (1.097 - 6.652) | 0.03 | 0.781  (0.305 - 1.999) | 0.61 | 0.865  (0.258 - 2.901) | 0.81 |
| Hyperuricemia/ gout* | NA | NA | NA | NA | 1.506  (0.307 - 7.378) | 0.61 | NA | NA |
| Cardiomyopathies* | 1.573  (0.273 - 9.077) | 0.61 | 0.882  (0.197 - 3.952) | 0.87 | 2.152  (0.880 - 5.262) | 0.09 | 2.276  (0.929 - 5.576) | 0.07 |
| Valvular cardiopathies* | 1.058  (0.203 - 5.504) | 0.95 | 0.684  (0.342 - 1.370) | 0.28 | 0.572  (0.338 - 0.968) | 0.04 | 0.717  (0.352 - 1.460) | 0.36 |
| Hypertensive cardiopathies* | 2.494  (0.801 - 7.767) | 0.11 | 0.649  (0.250 - 1.687) | 0.38 | 1.010  (0.491 - 2.074) | 0.98 | 0.647  (0.242 - 1.726) | 0.38 |
| Inflammatory cardiopathies* | NA | NA | 2.713  (1.167 - 6.308) | 0.02 | 0.646  (0.249 - 1.675) | 0.37 | 1.138  (0.340 - 3.810) | 0.83 |
| Rhythmogenic cardiopathies* | 1.676  (0.187 - 14.988) | 0.64 | 1.492  (0.462 - 4.817) | 0.50 | 1.466  (0.556 - 3.864) | 0.44 | 3.247  (1.040 - 10.137) | 0.04 |
| Ischemic heart disease* | 2.435  (0.742 - 7.989) | 0.14 | 1.016  (0.448 - 2.306) | 0.97 | 1.147  (0.641 - 2.054) | 0.64 | 1.112  (0.464 - 2.661) | 0.81 |
| Atherosclerosis* | NA | NA | 1.077  (0.093 - 12.512) | 0.95 | NA | NA | NA | NA |
| Pulmonary diseases* | NA | NA | NA | NA | NA | NA | NA | NA |
| Kidney disease* | NA | NA | 0.522  (0.120 - 2.272) | 0.39 | 1.785  (0.756 - 4.215) | 0.19 | 0.662  (0.088 - 4.991) | 0.69 |

**Supplementary Table 10:** Hazard ratio (HR) with corresponding 95% confidence interval (CI) for the outcomes myocardial infarction, embolic stroke or ICH and bleeding, other than ICH (from competing risk analyses). Covariables with NA (“not available” entries were not included to the model, since not enough data was available to compute the estimators and p-values. * Diagnosis before index OP


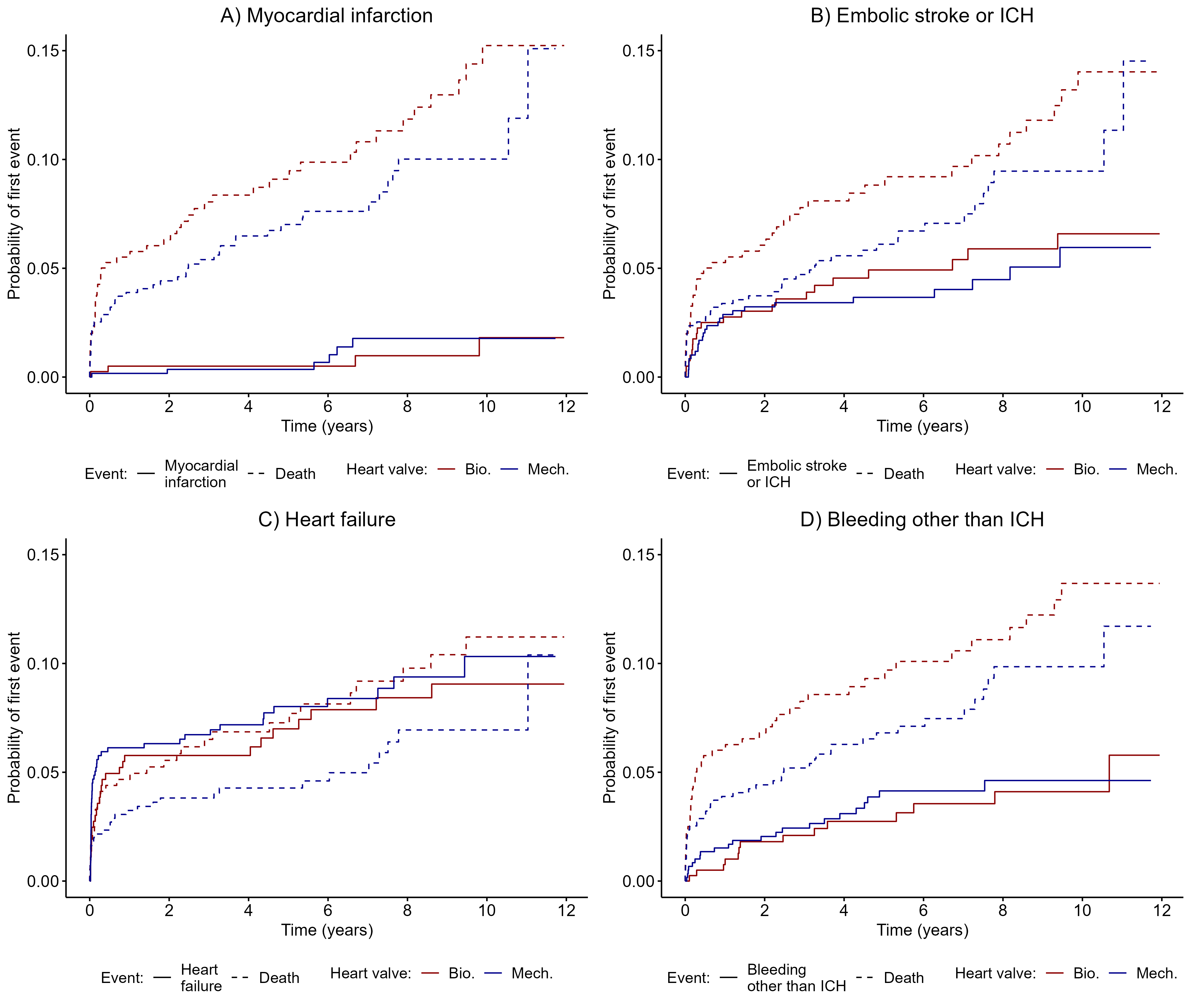


**Supplementary Figure 1:** Cumulative incidence curves for myocardial infarction (A), embolic stroke or ICH (B) and heart failure (C) and bleeding other than ICH (D) with competing event death).

9.2.) Outcome: Major Adverse Cardiac Events (MACE)

|  | MACE | |
| --- | --- | --- |
|  | **HR (95% CI)** | **p-value** |
| Heart valve (sB-AVR) | 1.164 (0.867 - 1.562) | 0.31 |
| Age (per one year increase) | 1.007 (0.988 - 1.027) | 0.46 |
| Sex (female) | 1.077 (0.779 - 1.488) | 0.66 |
| Myocardial infarction* | 2.161 (0.888 - 5.260) | 0.09 |
| Embolic Stroke or ICH* | 2.274 (1.066 - 4.852) | 0.03 |
| Diabetes mellitus* | 2.175 (1.097 - 4.311) | 0.03 |
| Adiposity* | 1.198 (0.599 - 2.394) | 0.61 |
| Hyperlipidemia* | 1.176 (0.696 - 1.990) | 0.54 |
| Hyperuricemia/gout* | 0.406 (0.051 - 3.241) | 0.40 |
| Cardiomyopathies* | 2.590 (1.529 - 4.390) | <0.001 |
| Valvular cardiopathies* | 0.691 (0.501 - 0.953) | 0.02 |
| Hypertensive cardiopathies* | 0.934 (0.608 - 1.434) | 0.75 |
| Inflammatory cardiopathies* | 1.791 (1.085 - 2.955) | 0.02 |
| Rhythmogenic cardiopathies* | 0.998 (0.512 - 1.944) | 0.99 |
| Ischemic heart disease* | 1.141 (0.763 - 1.706) | 0.52 |
| Atherosclerosis* | 2.928 (1.038 - 8.262) | 0.04 |
| Pulmonary diseases* | 0.694 (0.165 - 2.914) | 0.62 |
| Kidney disease* | 1.509 (0.838 - 2.718) | 0.17 |

**Supplementary Table 11:** Hazard ratios (HR) with corresponding 95% confidence interval (CI) and p-values for the outcome MACE (from Cox regression). * Diagnosis before index OP


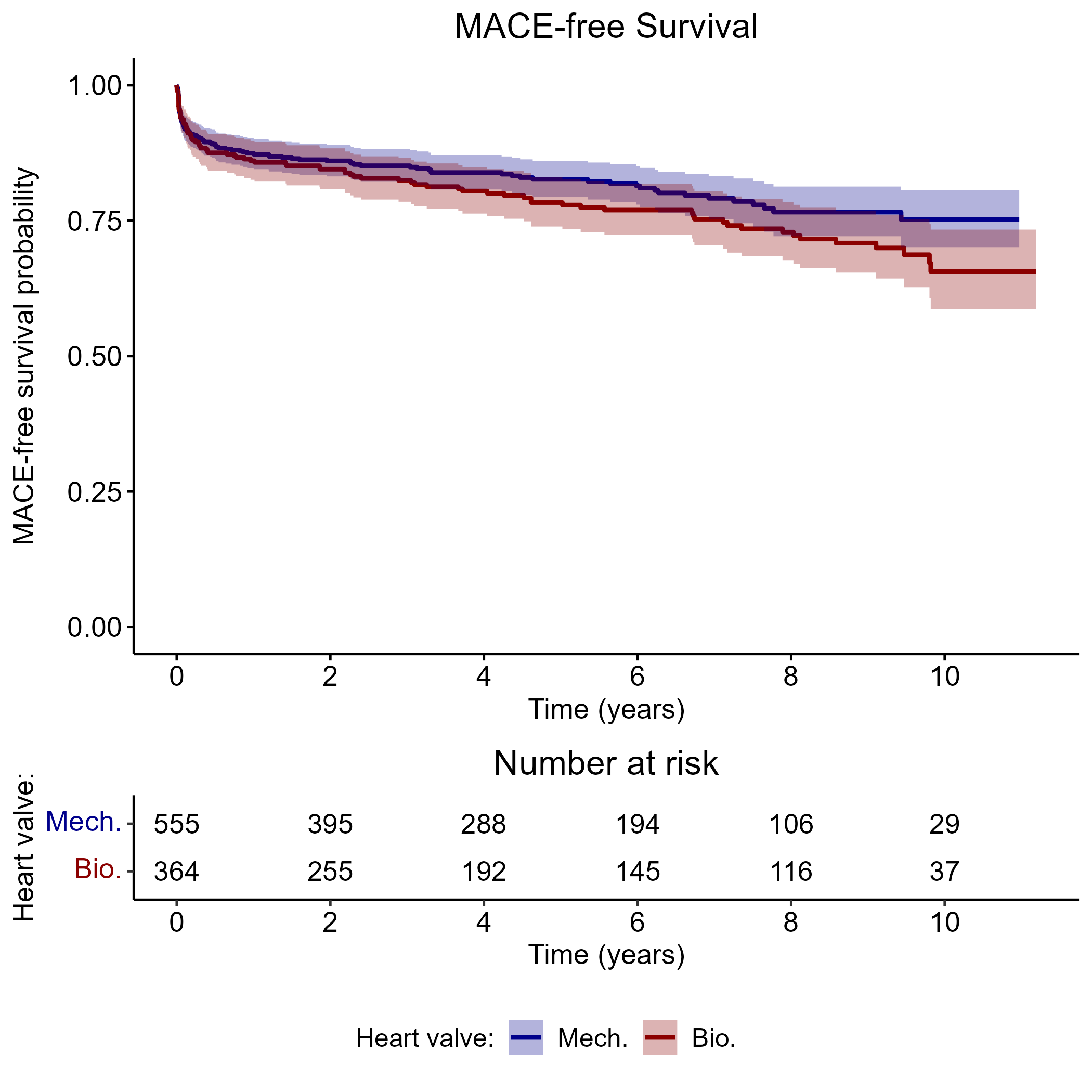


**Supplementary Figure 2:** Kaplan-Meier curves for MACE-free survival.

1. **Event probabilities for outcomes**

|  |  |  | Years | | | |
| --- | --- | --- | --- | --- | --- | --- |
| Event | Group |  | 2 | 5 | 7 | 10 |
| Overall Death | sM-ARV | Number at Risk | 535 | 359 | 240 | 87 |
|  |  | Event Probability | 0.044 | 0.068 | 0.083 | 0.104 |
|  |  | 95% CI | 0.027 - 0.060 | 0.047 - 0.089 | 0.058 - 0.107 | 0.074 - 0.134 |
|  | sB-ARV | Number at Risk | 347 | 241 | 192 | 111 |
|  |  | Event Probability | 0.068 | 0.094 | 0.111 | 0.157 |
|  |  | 95% CI | 0.043 - 0.092 | 0.064 - 0.123 | 0.077 - 0.143 | 0.111 - 0.201 |
| Reoperation or Death | sM-ARV | Number at Risk | 469 | 275 | 172 | 38 |
|  |  | Event Probability | 0.055 | 0.089 | 0.105 | 0.124 |
|  |  | 95% CI | 0.037 - 0.074 | 0.064 - 0.114 | 0.075 - 0.134 | 0.088 - 0.158 |
|  | sB-ARV | Number at Risk | 399 | 300 | 209 | 156 |
|  |  | Event Probability | 0.095 | 0.140 | 0.163 | 0.262 |
|  |  | 95% CI | 0.066 - 0.124 | 0.102 - 0.175 | 0.121 - 0.203 | 0.191 - 0.327 |
| Reoperation | sM-ARV | Number at Risk | 470 | 276 | 173 | 39 |
|  |  | Event Probability | 0.012 | 0.025 | 0.030 | 0.030 |
|  |  | 95% CI | 0.005 - 0.024 | 0.013 - 0.042 | 0.016 - 0.051 | 0.016 - 0.051 |
|  | sB-ARV | Number at Risk | 301 | 210 | 157 | 45 |
|  |  | Event Probability | 0.039 | 0.057 | 0.062 | 0.107 |
|  |  | 95% CI | 0.023 - 0.062 | 0.036 - 0.085 | 0.039 - 0.092 | 0.067 - 0.158 |
| Myocardial Infarction | sM-ARV | Number at Risk | 518 | 330 | 213 | 71 |
|  |  | Event Probability | 0.004 | 0.004 | 0.018 | 0.018 |
|  |  | 95% CI | 0.001 - 0.012 | 0.001 - 0.012 | 0.007 - 0.038 | 0.007-0.038 |
|  | sB-ARV | Number at Risk | 335 | 235 | 181 | 92 |
|  |  | Event Probability | 0.005 | 0.005 | 0.010 | 0.018 |
|  |  | 95% CI | 0.001-0.017 | 0.001 -0.017 | 0.003 - 0.028 | 0.005 - 0.047 |
| Embolic Stroke or ICH | sM-ARV | Number at Risk | 506 | 320 | 207 | 69 |
|  |  | Event Probability | 0.032 | 0.037 | 0.040 | 0.060 |
|  |  | 95% CI | 0.020 - 0.049 | 0.023-0.054 | 0.026 - 0.060 | 0.036 - 0.092 |
|  | sB-ARV | Number at Risk | 328 | 226 | 176 | 90 |
|  |  | Event Probability | 0.030 | 0.049 | 0.054 | 0.066 |
|  |  | 95% CI | 0.017 -0.051 | 0.030 - 0.075 | 0.033 - 0.082 | 0.041 - 0.099 |
| Heart failure | sM-ARV | Number at Risk | 456 | 288 | 196 | 64 |
|  |  | Event Probability | 0.063 | 0.080 | 0.08389 | 0.10321 |
|  |  | 95% CI | 0.045 - 0.086 | 0.059 - 0.106 | 0.061 - 0.111 | 0.073 - 0.139 |
|  | sB-ARV | Number at Risk | 290 | 200 | 154 | 79 |
|  |  | Event Probability | 0.058 | 0.067 | 0.079 | 0.091 |
|  |  | 95% CI | 0.037 -0.085 | 0.046 - 0.101 | 0.052-0.112 | 0.061 - 0.128 |
| Bleeding other than ICH | sM-ARV | Number at Risk | 508 | 316 | 208 | 70 |
|  |  | Event Probability | 0.020 | 0.041 | 0.041 | 0.046 |
|  |  | 95% CI | 0.011 – 0.034 | 0.026 – 0.062 | 0.026 – 0.062 | 0.029 – 0.070 |
|  | sB-ARV | Number at Risk | 329 | 227 | 173 | 90 |
|  |  | Event Probability | 0.018 | 0.027 | 0.036 | 0.041 |
|  |  | 95% CI | 0.008 – 0.035 | 0.014 - 0.048 | 0.019 – 0.060 | 0.022 – 0.068 |
| MACE | sM-ARV | Number at Risk | 395 | 231 | 147 | 29 |
|  |  | Event Probability | 0.140 | 0.174 | 0.209 | 0.248 |
|  |  | 95% CI | 0.110 - 0.168 | 0.139 - 0.207 | 0.168 - 0.248 | 0.194 - 0.299 |
|  | sB-ARV | Number at Risk | 255 | 173 | 130 | 37 |
|  |  | Event Probability | 0.155 | 0.217 | 0.247 | 0.344 |
|  |  | 95% CI | 0.116 - 0.192 | 0.170 - 0.261 | 0.196 - 0.296 | 0.267 - 0.413 |
| Death after Reoperation | sM-ARV | Number at Risk | 9 | 4 | 2 | 1 |
|  |  | Event Probability | 0.308 | 0.308 | 0.308 | 0.308 |
|  |  | 95% CI | 0.005 - 0.518 | 0.005 - 0.518 | 0.005 - 0.518 | 0.005 - 0.518 |
|  | sB-ARV | Number at Risk | 16 | 9 | 6 | 2 |
|  |  | Event Probability | 0.187 | 0.187 | 0.187 | 0.349 |
|  |  | 95% CI | 0.025 - 0.322 | 0.025 - 0.322 | 0.025 - 0.322 | 0.000 - 0.595 |

**Supplementary Table 12:** 2, 5, 7 and 10 year event probabilities and corresponding 95% CI for overall death, reoperation, myocardial infarction, embolic stroke or ICH, heart failure, bleeding other than ICH, MACE as well as death after reoperation.
