# Supplementary figures and images for: Revisiting aortic valve prosthesis choice in patients younger than 50 years: 10 years results of the AUTHEARTVISIT study
Source: Eur J Cardiothorac Surg. 2023 Sep 26;65(1):ezad308. doi: 10.1093/ejcts/ezad308 (PMC10761203; doi:10.1093/ejcts/ezad308)

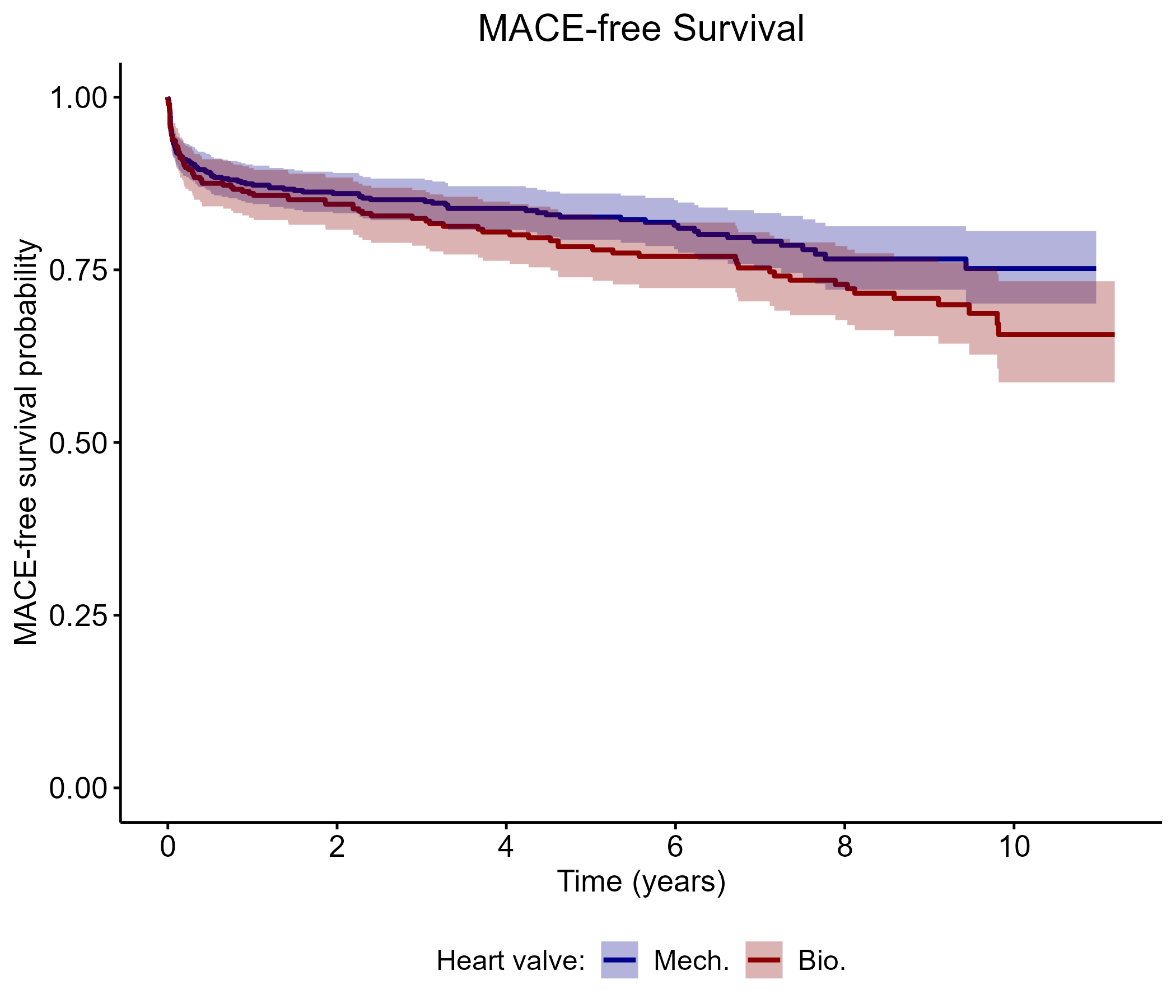

Supplement: ezad308_Supplementary_Data [file ezad308_supplementary_data.zip › Fig2_supp.tiff]

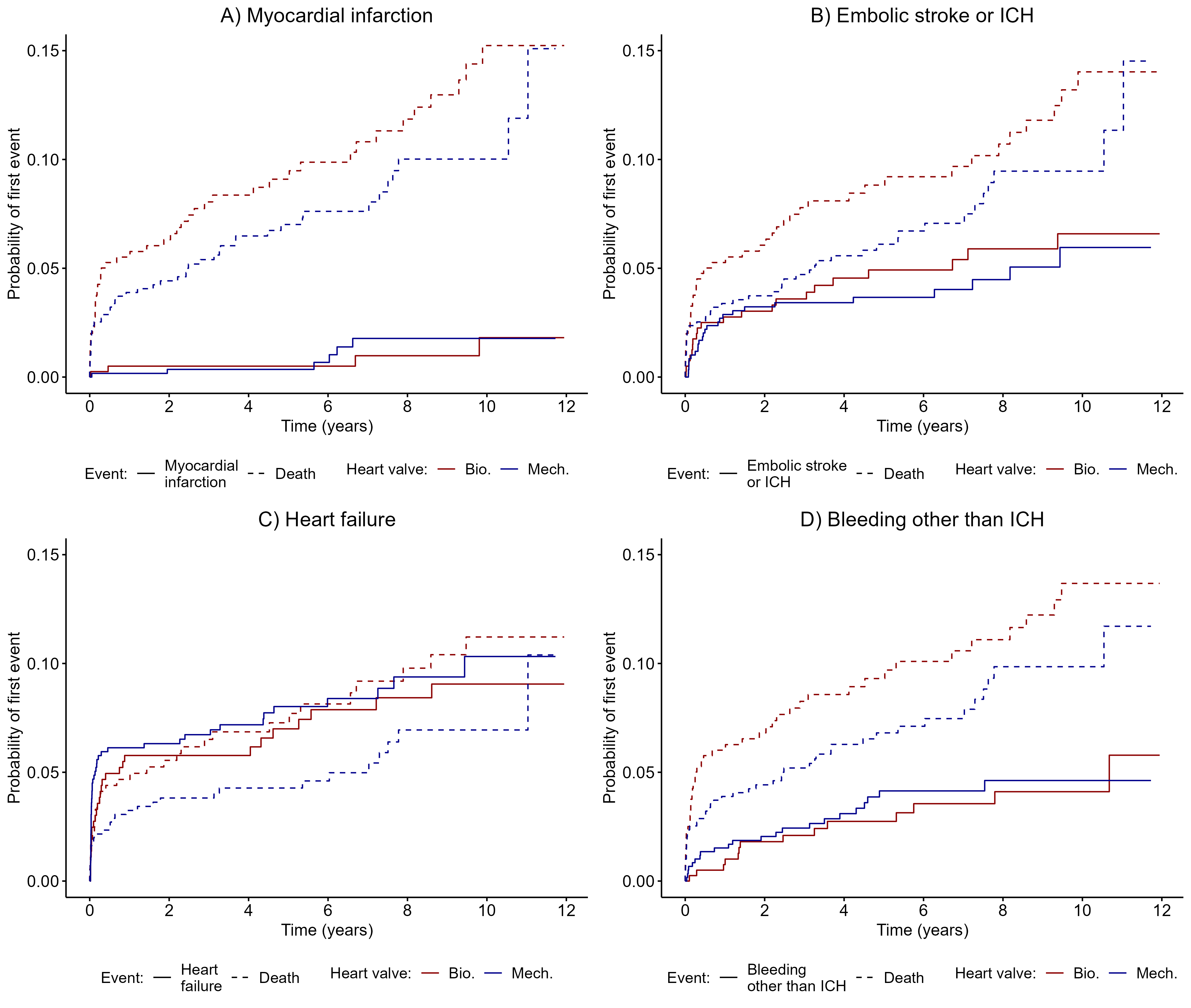

Supplement: ezad308_Supplementary_Data [file ezad308_supplementary_data.zip › Fig1_supp_rev.tiff]
